# Methylation Status of miR-34a and miR-126 in Non-Small Cell Lung Cancer (NSCLC) Tumor Tissues

**DOI:** 10.61186/ibj.3845

**Published:** 2023-10-14

**Authors:** Nazanin Mehrzad, Mohammad Saber Zamani, Amirabbas Rahimi, Masoud Shamaei, Morteza Karimipoor

**Affiliations:** 1 Molecular Medicine Department, Biotechnology Research Center, Pasteur Institute of Iran, Tehran, Iran;; 2 Mesih Deneshvari Hospital Shahid Beheshti Medical Sciences University, Tehran, Iran

**Keywords:** DNA methylation, miR-34a, miR-126, Non-small cell lung carcinoma

## Abstract

**Background:**

MiR-34a and miR-126 mainly act as tumor suppressors and are often downregulated in various cancers, including NSCLC. We aimed to determine the methylation status of miR-34a and miR-126 in NSCLC patients.

**Methods:**

The current study included 63 paraffin-embedded NSCLC and paired adjacent normal tissues. After DNA extraction and bisulfite treatment, the methylation status of miR-34a and miR-126 were evaluated using the MSP method.

**Results:**

There was no statistically significant difference between tumor and normal tissues regarding the methylation status of miR-34a and miR-126 (*p* > 0.05). Moreover, we found no significant correlation between the methylation status of miR-34a and miR-126 with patients’ demographic parameters, including gender, age, and pathology subtype (*p* > 0.05).

**Conclusion:**

Considering the low expression of mir-126 and mir-34 in NSCLC, more sensitive methods are recommended to be exploited for detecting the level of methylation or underlying mechanisms other than promoter hypermethylation in silencing these genes in NSCLC.

## INTRODUCTION

Lung cancer is the leading cause of cancer morbidity and mortality worldwide^[^^1^^]^. Histologically, this cancer is classified into two types: NSCLC and SCLC, which the former is the most common type and accounts for 80-85% of lung cancers. Recurrent loss-of-function mutations in tumor suppressor genes such as *TP53*, and also the aberrant expression of miRNA has frequently been found in NSCLC. 

MicroRNAs, small non-coding RNAs with an average 22 nucleotides in length, play a tumor suppressive or oncogenic role in various types of cancers. These genes are recognized as prognostic markers and/or potential therapeutic targets in cancers. For instance, *miR-126*, located in the seventh intron of the EGFL7 on chromosome 9, has fundamental roles in angiogenesis and inflammation, and its expression decreases in colorectal, gastric, esophageal, and pancreatic cancers^[^^3^^]^. It has been shown that the miR-34 family suppresses tumor growth by inhibiting the processes that stimulate cancer development. The emerging role of miR-34a has been revealed in cancer cell response to chemotherapy agents and treatment resistance. It has also been reported that miR-34a is downregulated in tumor cells due to 1p36 deletion (in neuroblastoma) or methylation of CpG islands in its promoter, particularly in breast, lung, kidney, and bladder cancers^[^4^]^. Altogether, miR-34a and miR-126 often have tumor-suppressive functions, and their expressions could be reduced through epigenetic mechanisms such as promoter hypermethylation. 

According to existing evidence on decreasing expression of miR-34a and miR-126 in lung cancer, and the biological and pathological significance of these two miRNAs, we hypothesized that their promoter is hypermethylated in NSCLC tumor tissues compared to normal tissues. Therefore, the present study aimed to evaluate the methylation status of mir-34a and mir-126 in NSCLC patients.

## MATERIALS AND METHODS

**Sample collection**

In the study, a total of 63 FFPE *blocks* of NSCLC and matched adjacent normal tissues were gathered from Masih Daneshvari Hospital, Tehran, Iran. Patients' clinical information is summarized in Table 1.

***DNA extraction and real-time PCR***

Genomic DNA from FFPE samples was extracted utilizing a DNA extraction kit (Yekta Tajhiz Azma, Iran) following the manufacturer's instructions. Qualitative and quantitative analyses of the extracted DNA was performed using a spectronanophotometer (BioTek, USA) and running on 1% agarose gel electrophoresis. Additionally, a PCR on a fragment of *GAPDH* gene was performed and electrophoresed on an agarose gel to confirm the integrity of DNA.

***Bisulfite treatment of genomic DNA and MSP***

*Bisulfite conversion of genomic DNA was performed using a EZ DNA Methylation-Gold Kit (Zymo Research, USA) according to the manufacturer’s instructions. Briefly, 2 μg of genomic DNA was treated with sodium bisulfite. The sequence of CpG island was predicted using the UCSC Genome Browser (https://genome.ucsc.edu/) and MethPrimer (https:// www.urogene.org/methprimer/). MSP was used to evaluate the CpG island methylation status in mir-126 and mir-34. In this context, bisulfite-treated DNAs were amplified using MSP primers specific for the methylated (M) and unmethylated (U) DNA, respectively. The primer sequences provided were as follows: *F: 5'- TTGGTTTTGGGTAGGTGTGTTTT-3' and R: 5'-AATCCTCATCCCCTTCACCACCA-3' *for *miR-34U*, F: *5'-GGTTTTGGGTAGGCGCGTTTC-3' and R: 5'-TCCTCATCCCCTTCACCGCCG-3' for miR-34M, *F: *5'-GTGGTGGTGGTGTGTGTGTGTTT-3' and R: 5'-CTCAACCCAACCCAAACAACAACCA-3' for miR-126U, and *F: *5'-GCGGCGCGTGCGCG TTT-3' and R: 5'-CCAACCCGAACGACGACCG-3' for miR-126M. *Moreover, DNA extracted from a blood sample was used as unmethylated control, and enzymatically treated DNA with CpG methyltransferase (M. SssI; Thermo Fisher Scientific, USA) was used as fully methylated genomic DNA. The MSP reaction was performed in a total volume of 20 μl. *To evaluate the sensitivity of MSP, serial dilutions of template DNA was prepared with different ratios of methylated and unmethylated control DNA. The density of the PCR product bands were visualized and analyzed by ImageJ software. The reactions were carried out for methylated and unmethylated primers in two different tubes. PCR products were electrophoresed on 2% agarose gel and visualized under UV illumination.

**Table 1:** Association of *methylation status of miR-126 and miR-34a *with patients’ clinical characteristics

***Characteristics***	***miR-126***				***miR-34a***		
	***Methylated*** ***N (%)***	***Unchanged*** ***N (%)***	**p** ***value***		***Methylated*** ***N (%)***	***Unchanged*** ***N (%)***	**p** ***value***
*Gender* *Male* *Female*			*0.416*				
	*16 (36.4)*	*28 (63.6)*			*5 (83.3)*	*39 (16.7)*	*0.452*
	*9 (47.4)*	*10 (52.6)*			*1 (31.6)*	*18 (68.4)*	
							
*Age group* *<50* *≥50*			*0.194*				
	*9 (52.9)*	*8 (47.1)*			*2 (11.8)*	*15 (88.2)*	*0.714*
	*16 (34.8)*	*30 (65.2)*			*4 (8.7)*	*42 (91.3)*	
							
*Involved pulmonary lobe* *Lower left* *Lower right* *Upper left* *Upper right*			*0.405*				
	*10 (40.0)*	*15 (60.0)*			*5 (20.0)*	*20 (80.0)*	*0.274*
	*10 (50.0)*	*10 (50.0)*			*1 (5.0)*	*19 (95.0)*	
	*2 (33.3)*	*4 (66.7)*			*0 (0.0)*	*6 (100.0)*	
	*0 (0.0)*	*3 (100.0)*			*0 (0.0)*	*3 (100.0)*	
							
*Tumor stage* *IB* *IIA* *IIB* *IIIA* *IIIB*			*< 0.0001*				
	*0 (0.0)*	*3 (100.0)*			*1 (33.3)*	*2 (66.7)*	*0.247*
	*5 (55.6)*	*4 (44.4)*			*2 (22.2)*	*7 (77.8)*	
	*0 (0.0)*	*23 (100.0)*			*2 (8.7)*	*21 (91.3)*	
	*10 (83.3)*	*2 (16.7)*			*0 (0.0)*	*12 (100.0)*	
	*3 (60.0)*	*2 (40.0)*			*0 (0.0)*	*5 (100.0)*	
							
*TNM* *TXN0M0* *TXNXM0* *TXNXMX*			*0.530*				
	*9 (37.5)*	*15 (62.5)*			*2 (8.3)*	*22 (91.7)*	*0.813*
	*10 (40.0)*	*15 (60.0)*			*3 (12.0)*	*22 (88.0)*	
	*0 (0.0)*	*2 (100.0)*			*0*	*2 (100.0)*	
							
*Tumor pathology* *AD* *SCC*			*0.005*				
	*20 (51.3)*	*19 (48.7)*			*3 (7.7)*	*36 (92.3)*	*0.626*
	*2 (11.8%)*	*15 (88.2%)*			*2 (11.8)*	*15 (88.2)*	

**Statistical analysis**

An IBM SPSS 23.0 statistics software was employed to analyze the obtained data. In descriptive statistics, mean and standard deviation were used for quantitative and percentage, and frequency was utilized for qualitative data. In the assessment of the data, the chi-square (χ2) and Wilcoxon signed-rank tests were applied to compare the categorical data and intragroup comparisons, respectively. *p* values less than 0.05 were considered as statistically significant. 

**Fig. 1:**
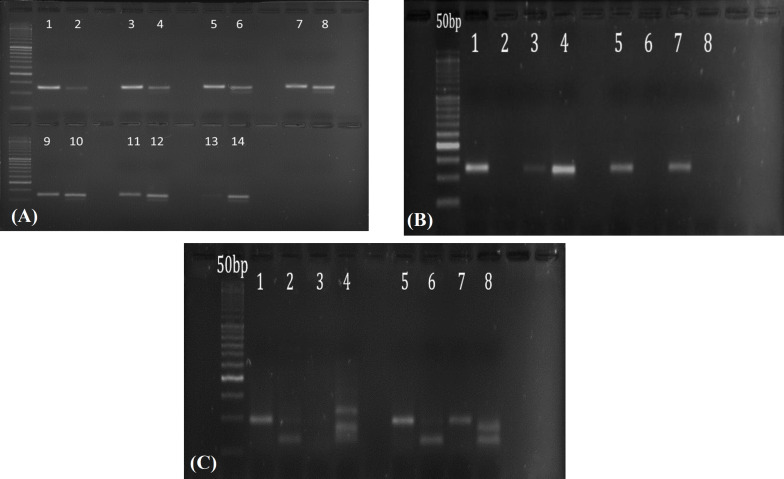
Evaluation of MSP method sensitivity for mir-34a CpG island. (A) Lanes 1 and 2: a ratio of 1:99 (1% fully methylated DNA plus 99% unmethylated DNA); lanes 3 and 4: a ratio of 5:95; lanes 5 and 6: a ratio of 10:90; lanes 7 and 8: a ratio of 20:80; lanes 9 and 10: ratio of 35:65; lanes 11 and 12: a ratio of 60:40; lanes 13 and 14: a ratio of 100:0. *(B). Electrophoresis of MSP products for miR-34a. Lanes 1 (unmethylated DNA plus miR-34U) and 2 (unmethylated DNA plus miR-34M) are negative controls; lanes 3 (methylated DNA plus miR-34U) and 4 (methylated DNA plus miR-34M) are positive controls; lanes 5 and 6 are related to the unmethylated and methylated primers of miR-34a in DNA samples of normal tissues, respectively; lanes 7 and 8 are related to the unmethylated and methylated primers of miR-34a in DNA samples of normal tissues, respectively. A 50 bp DNA ladder was utilized according to the length of the products. (C) Electrophoresis picture of MSP for miR-126. Lanes 1 (unmethylated DNA plus miR-126U) and 2 (unmethylated DNA plus miR-126M) are negative controls; lanes 3 (methylated DNA plus miR-126U) and 4 (methylated DNA plus miR-126M) are positive controls (in lane 4, the upper band is non-specific); lanes 5 and 6 are related to the unmethylated and methylated primers of miR-126 in DNA samples of normal tissues, respectively; lanes 7 and 8 are related to the unmethylated and methylated primers of miR-126 in DNA samples of normal tissues, respectively (in lanes 6 and 8, the bottom band is non-specific). A 50 bp DNA ladder was utilized according to the length of the products*

## RESULTS

In this study, the methylation status of CpG islands of mir-34a and mir-126 promoters in 63 FFPE of NSCLC and paired adjacent normal tissues were investigated by MSP. The mean age of the patients at the diagnosis time was 54 ± 12.54. For sensitivity assessment of the MSP method, a serial dilution of methylated and unmethylated control DNAs with different ratios was amplified with methylated and unmethylated miR-34a primers in parallel. According to the results, the MSP technique showed a sensitivity between 1-5% for distinguishing methylated from unmethylated miR-34a DNA (Fig. 1A). Using optimized MSP method, we investigated the methylation status of the samples for mir-34a and mir-126. The results of MSP reaction for miR-34a and miR-126 are presented in Figure 1B and 1C, respectively. 

The methylation status of miR-34a and miR-126 in NSCLC tumor samples did not show significant differences (*p* > 0.05) as compared to the adjacent normal tissues. *The methylation status of the two microRNAs in the above-mentioned samples were examined along with the parameters of sex, pathology subtype of NSCLC, age, and tumor staging by Chi-square test. As the results depicted in *Table 1, the methylation status of miR-126 was significantly related to tumor stage (*p* < 0.0001) and tumor pathology (*p* = 0.005). However, the patient's gender, age, involving pulmonary lobe, and TNM were not significantly correlated with the methylation status of miR-126 (*p* > 0.05). There were also no significant differences between the methylation status of the *miR-34a *gene and the patient's sex and age, tumor stage, involving pulmonary lobe, TNM, and tumor pathology (*p* > 0.05; Table 1).

**Fig. 2:**
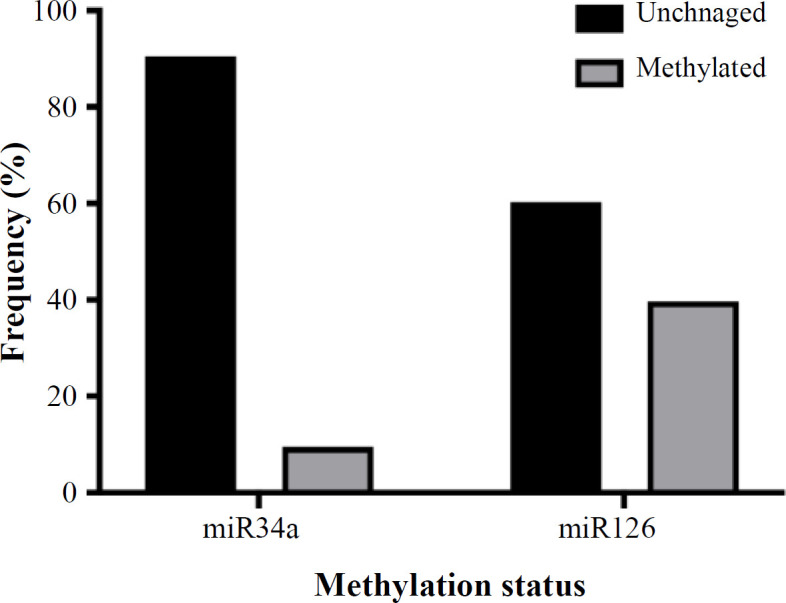
Evaluation of methylation status of miR-34a and miR-126 genes in NSCLC patients

The frequency of miR-34a methylation in 63 NSCLC tissues showed that miR-34a was unmethylated in 90.5% of the samples and methylated in the remaining (9.5%). Also, miR-126 was unmethylated in 60.3% of the specimens and methylated in 39.7% (Fig. 2).

## DISCUSSION

Epigenetic changes have pivotal role in cancer initiation and progression. In addition to DNA methylation and histone modifications, miRNAs play an important role in post-transcriptional regulation of protein-coding genes. The regulation of miRNA expression is affected by CpG island methylation, copy number variations, chromosomal translocations, and somatic mutations^[^^5^^]^.

miR-34 expression is mostly regulated by the transcription factors and methylation status of CpG islands. Previous studies have shown that miR-34a is suppressed in different cancers through various molecular mechanisms, including deletion^[^^5^^]^. According to a study by Garofalo et al., it has been indicated that miR-34a induces apoptosis in lung cancer cells by targeting PDGFR-α/β^[^^6^^]^. In another study, the miR-34a expression has been demonstrated to prevent metastasis in NSCLC by inhibiting the growth of cancer stem cells^[^^7^^]^. P53 directly activates the expression of miR-34a, leading to the dramatic inhibition of cell-cycle progression, proliferation, apoptosis induction, and DNA repair, as well as suppresses the angiogenesis. Indeed, P53 is activated in response to DNA damage and cellular stress, which increases miR-34a expression. It has been described that miR-34a and P53 positively affect the expression of each other^[^^7^^]^. *TAp73 *and *ELK1* are also positive regulators for miR-34a expression^[^^8^^]^. Epigenetic silencing of genes by CpG islands methylation in the promoter is a fundamental mechanism of tumor suppressor gene inactivation^[^^9^^]^. Hypermetylation of CpG sites in miR-34a promoter has been documented in solid tumors, including breast, colon, lung, bladder, kidney, and pancreas cancers and could serve as a prognostic and/or therapeutic biomarker^[^^10^^]^. For instance, the exposure of tumor cells to 5-aza-2'deoxycytidine, an inhibitor of DNA methyltransferase, significantly reduces the methylation level in CpG regions and reactivates miR-34a expression^[^^8^^]^. 

The current study investigated the methylation status of miR-34a and miR-126 in 63 paired NSCLC tumor samples using the MSP technique. Methylation of miR-34a and miR-126 was observed in 9.5% and 39.7% of NSCLC specimens, respectively. The MSP method had a sensitivity of about 1-5% for the methylation of these genes and could detect the methylation status of all DNA samples (methylated or unmethylated). However, MSP method can lead to false-positive results, if it is not completely optimized. Importantly, it is inevitable to optimize the MSP with positive and negative controls. As the results shown in the present study, the methylation status of miR-34a in NSCLC tissues was similar to the adjusted normal tissues, suggesting the involvement of other mechanisms. 

MiR-126 alters the biological functions of some genes such as *PI3K*, *EGFL7*, *HOXA9*, *sKRAS*, *CRK*, *ADAM9*, *IRS-1*,* SOX-2*,* SLC7A5*, and *VEGF*, as well as involves in inflammation, cell migration, angiogenesis, recurrence of cancer, and metastasis^[^^11^^]^. In a study conducted by Zhu et al., it has been exhibited that the expression of miR-126 decreases in NSCLC patients compared to normal subjects^[^^12^^]^. Moreover, it has been found that miR-126 reduces angiogenesis and proliferation of NSCLC by targeting VEGF^[^^13^^]^. On the other hand, it has been shown that mir-126 decreases proliferation and induces apoptosis in NSCLC by activating STAT3 and caspase-3 expression^[^^14^^]^. MiR-126 is reduced in cancers by several mechanisms, including epigenetic modifications. Li et al. have indicated that the decreased expression of miR-126 in AML is associated with its methylation. Using the bisulfite genomic sequencing, they examined the miR-126 methylation status in 18 specimens consisting of 8 CBF AML, 10 non-CBF leukemia, and three control samples. Methylation of CpG islands of this miRNA was observed in 70.8% of CBF AML and 94.4% of non-CBF leukemia cases^[^^15^^]^. Zhang and coworkers have demonstrated that miR-126 is downregulated through the methylation of its host gene, EGFL7, in breast cancer^[^^16^^]^. This negative correlation between the methylation of miR-126 and its expression has also been confirmed in colorectal cancer^[^^13^^]^. Regarding the results of the present study, the methylation status of miR-126 in NSCLC samples has been found in 39.7% of the tumor tissue samples. It is noteworthy that the negative control samples in our study were the adjacent normal tissues of the same patients. The epigenetic alteration in normal tissue around the tumor may be occurred, despite the normal morphology of the cells^[^^13^^]^.

## CONCLUSION

Methylation of miR-34a and miR-126 were found in 9.5% and 39.7% of NSCLC samples, respectively. Given the methylation form of mir-34a in normal and cancerous samples with no significant difference, other mechanisms may be involved in reducing the miR-34a expression in NSCLC.

## DECLARATIONS

### Acknowledgments

 No AI-assisted technologies were used in the production of the present study.

### Ethical approval

All the experimental procedures in this study were conducted in accordance with the Helsinki Declaration of 1975 and approved by the Research Ethics Committee of Tehran University of Medical Sciences, Tehran, Iran (ethical code: IR.TUMS.SPH.REC. 1398.014) and *the Ethics committee of Masih Daneshvari Hospital, Tehran, Iran (ethical code: sbmul.REC1394.112).*

### Consent to participate

All the subjects included in the study voluntarily agree to participate in this research. Written informed consents were provided by all the subjects who participated in the study.

### Consent for publication

All authors reviewed the results and approved the final version of the manuscript.

### Authors’ contributions

NM: contributing to experimental work and drafting the manuscript; MSZ: performing the experimental works and data analysis; AR: contributing to sampling and data gathering; MS: providing sample and clinical data; MK: designing the study, supervising the experimental works, and editing the final version of manuscript.

### Data availability

All the data used to support the findings of this study can be found within the manuscript.

### Competing interests

The authors declare that they have no competing interests. 

### Funding

This research received no specific grant from any funding agency in the public, commercial, or not-for-profit sectors. 

### Supplementary information

The online version does not contain supplementary material.

## References

[1] Sung H, Ferlay J, Siegel RL, Laversanne M, Soerjomataram I, Jemal A (2021). Global cancer statistics 2020: GLOBOCAN estimates of incidence and mortality worldwide for 36 cancers in 185 countries. CA Cancer J Clin.

[2] Sussan TE, Pletcher MT, Murakami Y, Reeves RH (2005). Tumor suppressor in lung cancer 1 (TSLC1) alters tumorigenic growth properties and gene expression. Mol Cancer..

[3] Li ZH, Weng X, Xiong QY, Tu JH, Xiao A, Qiu W (2017). miR-34a expression in human breast cancer is associated with drug resistance. Oncotarget.

[4] Kalfert D, Ludvikova M, Pesta M, Ludvik J, Dostalova L, Kholová I (2020). Multifunctional roles of miR-34a in cancer: a review with the emphasis on head and neck squamous cell carcinoma and thyroid cancer with clinical implications. Diagnostics(Basel).

[5] Li X, Ren Z, Tang J (2014). MicroRNA-34a: a potential therapeutic target in human cancer. Cell Death Dis.

[6] Garofalo M, Jeon YJ, Nuovo GJ, Middleton J, Secchiero P, Joshi P (2013). MiR-34a/c-dependent PDGFR-α/β downregulation inhibits tumorigenesis and enhances TRAIL-induced apoptosis in lung cancer. Plos One.

[7] Zhang L, Liao Y, Tang L (2019). MicroRNA-34 family: a potential tumor suppressor and therapeutic candidate in cancer. J Exp Clin Cancer Res.

[8] Kwon H, Song K, Han C, Zhang J, Lu L, Chen W (2017). Epigenetic silencing of miRNA-34a in human cholangiocarcinoma via EZH2 and DNA methylation: impact on regulation of notch pathway. Am J Pathol.

[9] Cohen Y, Merhavi Shoham E, Avraham RB, Frenkel S, Pe'er J, Goldenberg Cohen N (2008). Hypermethylation of CpG island loci of multiple tumor suppressor genes in retinoblastoma. Exp Eye Res.

[10] Saito Y, Nakaoka T, Saito H (2015). microRNA-34a as a therapeutic agent against human cancer. J Clin Med.

[11] Rouigari M, Dehbashi M, Ghaedi K, Pourhossein M (2018). Targetome analysis revealed involvement of miR-126 in neurotrophin signaling pathway: a possible role in prevention of glioma development. Cell J.

[12] Zhu W, Zhou K, Zha Y, Chen D, He J, Ma H (2016). Diagnostic value of serum miR-182, miR-183, miR-210, and miR-126 levels in patients with early-stage non-small cell lung cancer. Plos One.

[13] Zhang Y, Wang X, Xu B, Wang B, Wang Z, Liang Y (2013). Epigenetic silencing of miR-126 contributes to tumor invasion and angiogenesis in colorectal cancer. Oncol Rep.

[14] Zhang Z, Wang J, Cheng J, Yu X (2018). Effects of miR-126 on the STAT3 signaling pathway and the regulation of malignant behavior in lung cancer cells. Oncol Lett.

[15] Li Z, Lu J, Sun M, Mi S, Zhang H, Luo RT (2008). Distinct microRNA expression profiles in acute myeloid leukemia with common translocations. Proc Natl Acad Sci U S A.

[16] Zhang Y, Yang P, Sun T, Li D, Xu X, Rui Y (2013). miR-126 and miR-126* repress recruitment of mesenchymal stem cells and inflammatory monocytes to inhibit breast cancer metastasis. Nat Cell Biol.

